# Supplementary material for: Climate-driven shifts in sediment chemistry enhance methane production in northern lakes
Source: Nat Commun. 2018 May 4;9:1801. doi: 10.1038/s41467-018-04236-2 (PMC5935729; doi:10.1038/s41467-018-04236-2)
Supplement: Supplementary file 1 — Supplementary Information [file 41467_2018_4236_MOESM1_ESM.pdf]

Supplementary Information

Climate-driven shifts in sediment chemistry enhance methane production in northern lakes

Emilson et al.

Supplementary Table 1: Comparison of total phenolic content of foliage from several common macrophytes and trees. Ranges or mean (SD) of fresh litter total phenolic content is shown in Gallic Acid Equivalents (GAE).

Species	Total Phenolics (mg/g GAE)	Study Location and Reference
**Macrophytes**		
Typha angustifolia	23.1 (2.5) *	USA [1]
	0 – 17.7	USA [2]
Typha latifolia	3.2 – 17.9	USA [2]
	8.8 (NA)	Poland [3]
	8.2 (0.1)	Finland [4]
Schoenoplectus tabernaemontani	0.2 – 12.4	USA [2]
Phragmites australis	8.7 – 18	USA [2]
	13.9 (3.1) *	China [5]
	7.0 (NA)	Poland [3]
	7.1 (1.6)	Nigeria [6]
	5.7 (0.3)	Finland [4]
**Coniferous**		
Picea mariana	25.3 (1.1)	Canada [7]
	55 (NA) [¥] **	Canada [8]
	43 (NA)	Canada [9]
Acer rubrum	118 – 132 *	Canada [10]
Acer saccharum	80 – 125 *	Canada [10]
**Deciduous**		
Betula papyrifera	138 (NA) [¥] **	Canada [8]
	69 – 85 *	Canada [10]
Populus tremuloides	108 (NA)	Canada [9]
	96 (NA)**	Canada [8]
	30 – 64 *	Canada [10]
Quercus rubra	83.4 (5) *	USA [11]
	73.5 – 126 *	USA [12]
	16 – 81 *	USA [10]
Quercus alba	129 (2.5) *	USA [11]
	50 – 83 *	USA [10]

* Results originally presented in Tannic Acid Equivalents (TAE), GAE estimated as 0.8253 X TAE as in ref. 1.

** Results originally presented in Catechin Equivalents (CE), GAE estimated as 0.8363 X CE as in ref. 1.

[¥] approximated visually from graph

Supplementary Table 2: PCR results for sulphate reducing bacteria (SRB). PCR for the *dsr*A gene could not successfully amplify any detectable SRBs, and SRB related reads were in low abundance in the 16S rRNA gene sequencing libraries for each sediment type. Results from amended sediments are presented as means (SDs) across duplicates from pooled samples of three OM percentages (10, 20, 40%), with two control replicates (see Methods). While pooling prevents comparison of variability within and among treatments, mean SRB numbers were consistently low (<1%) across treatments.

Sample	N	PCR (*dsr*A)	SRB 16S Reads *	Total 16S Reads	% SRB Abundance
CTR 1	1	-	132	16805	0.78
CTR 2	1	-	75	12263	0.61
CON	6	-	2.66 (1.75)	39745 (14331)	0.01 (0.00)
TYP	6	-	40.3 (4.53)	33979 (7213)	0.12 (0.05)
DEC	6	-	7.65 (3.64)	86774 (26655)	0.01 (0.01)

* Only six SRB genera were detected: *Desulfosporosinus, Desulfomonile, Desulfobacca, Desulfobulbus, Desulfococcus, Desulfovibrio*.

Supplementary Table 3: Production estimates from Boreal Shield lakes for a 150-day growing season. CO_2 equivalence assumes the conservative estimate of 1 kg CH_4 equals 25 kg CO_2 to keep consistent with ref. 2. Experimental variaton in CH_4 production estimates (standard error) are used to calculate range in total CH_4 emission, with uncertainty in lake area from five different climate models (GCMs) and three climate change scenarios (RCPs) propagated into the range for projected values (see text).

	Proportion of lakes with *T. latifolia* (probability > 0) *	Total lake area covered (km^2) **	Total CH_4 emission ($x10^{-3}$ Pg CO_2 equivalents)
Current	33%	5015	0.75 - 1.83
Projected	55 - 85%	8668 - 12403	1.30 - 4.53

* of 28043 boreal lakes between 0.1 and 1000 km^2, which excludes very large lakes, small ponds, rivers, and wetlands that are abundant in the Boreal Shield ecozone and may contribute substantially to additional macrophyte-related CH_4 emissions.

** sum of the product of probability of occurrence and 28% of lake area in each lake (see text)

Supplementary Figures

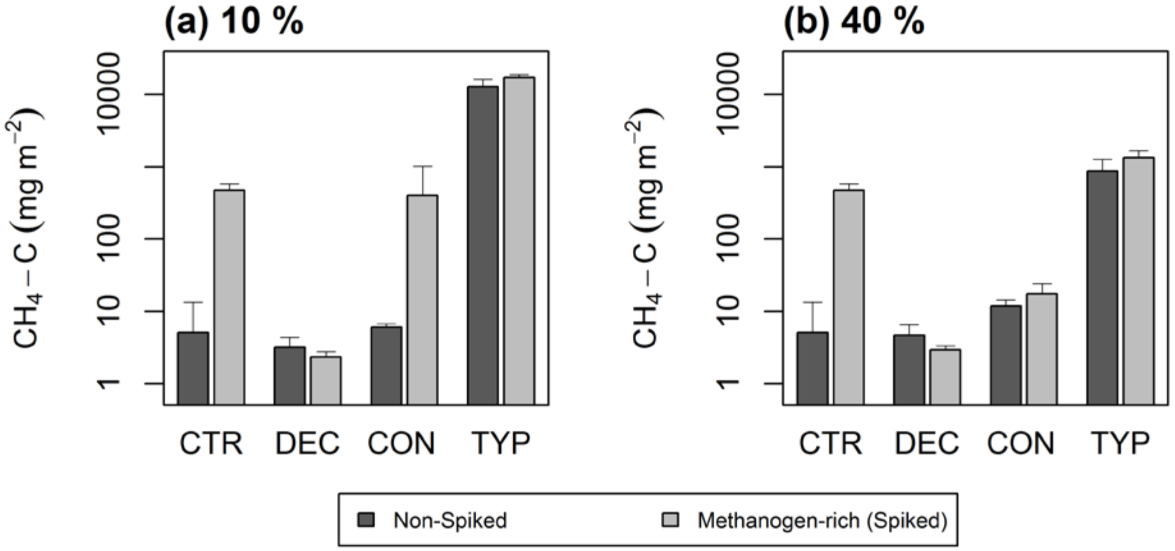

Supplementary Fig. 1: CH_4 production in 10 (A) and 40% (B) amendments. Patterns of production in 10 and 40% OM methanogen-rich (spiked) and non-spiked sediments over a 150-day growing season are comparable to those seen in 20% OM (in Fig. 1). There are n = 4 replicates per amendment type and results are shown on a log scale because of large differences between TYP and the other amendments. Error bars represent standard errors in production estimates.

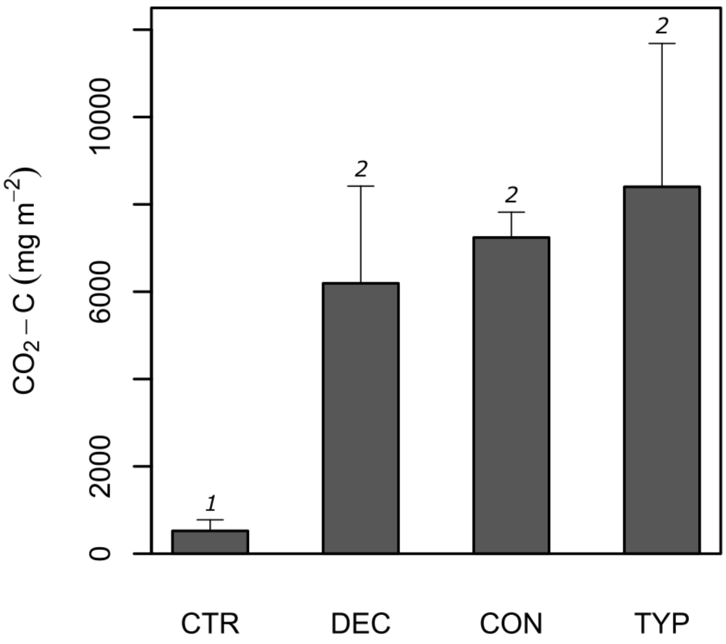

Supplementary Fig. 2: CO_2 production in amended sediments. There were no differences in CO_2 production over a 150-day growing season among 20% amended sediments. Different numbers (1,2) represent significant differences ($p < 0.05$) among amendments (ANOVA $F_{3, 12} = 12.12$). Error bars represent standard errors in production estimates.

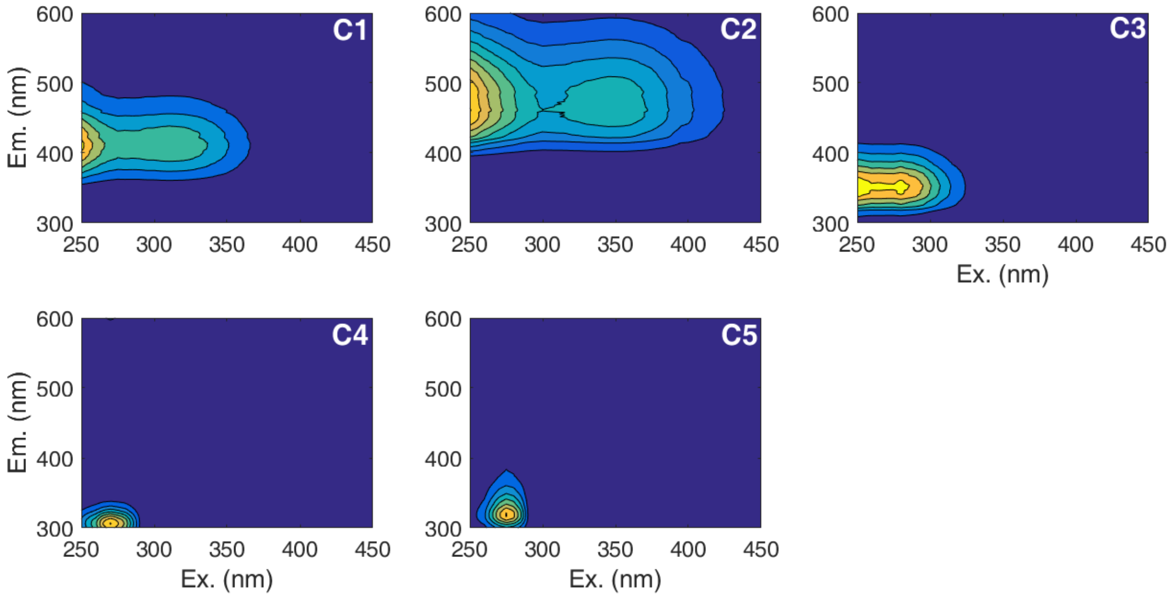

Supplementary Fig. 3: Five dissolved organic matter PARAFAC components identified in sediment porewater. Identified components included a phenol leachate protein-like component (C5), as well as two humic-like components (C1, C2), and tryptophan- and tyrosine-like components (C3 and C4 respectively).

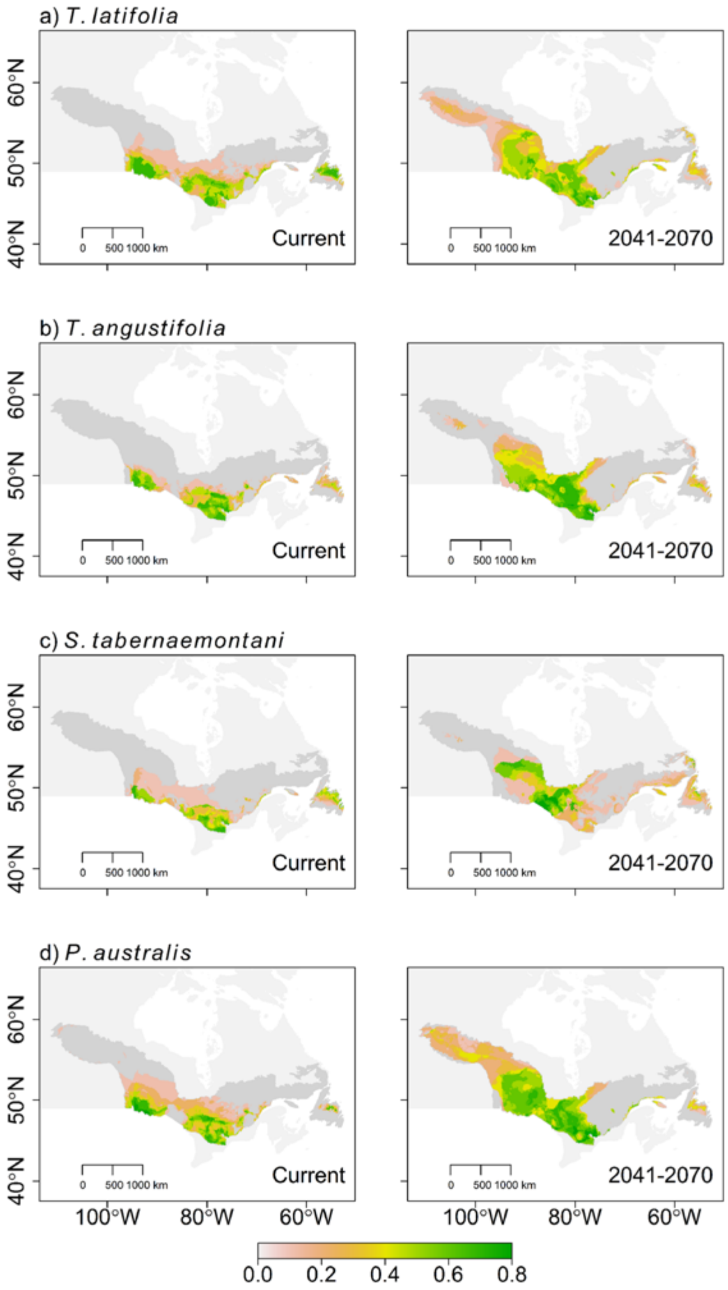

Supplementary Fig. 4: Distributions of four widespread emergent macrophytes are expected to increase in the Boreal Shield by 2041 to 2070. Increases are based on predicted shifts in climatic conditions from the RCP 4.5 emission scenario under the composite-AR5 climate model. Current and predicted probability of occurrence data are shown for four emergent macrophytes common to North American lakes: a) *Typha latifolia*, b) *Typha angustifolia*, c) *Schoenoplectus tabernaemontani*, and d) *Phragmites australis*. Data were obtained from Natural Resources Canada MaxEnt species distribution models generated using a climate envelope approach that incorporates extensive observational data [3].

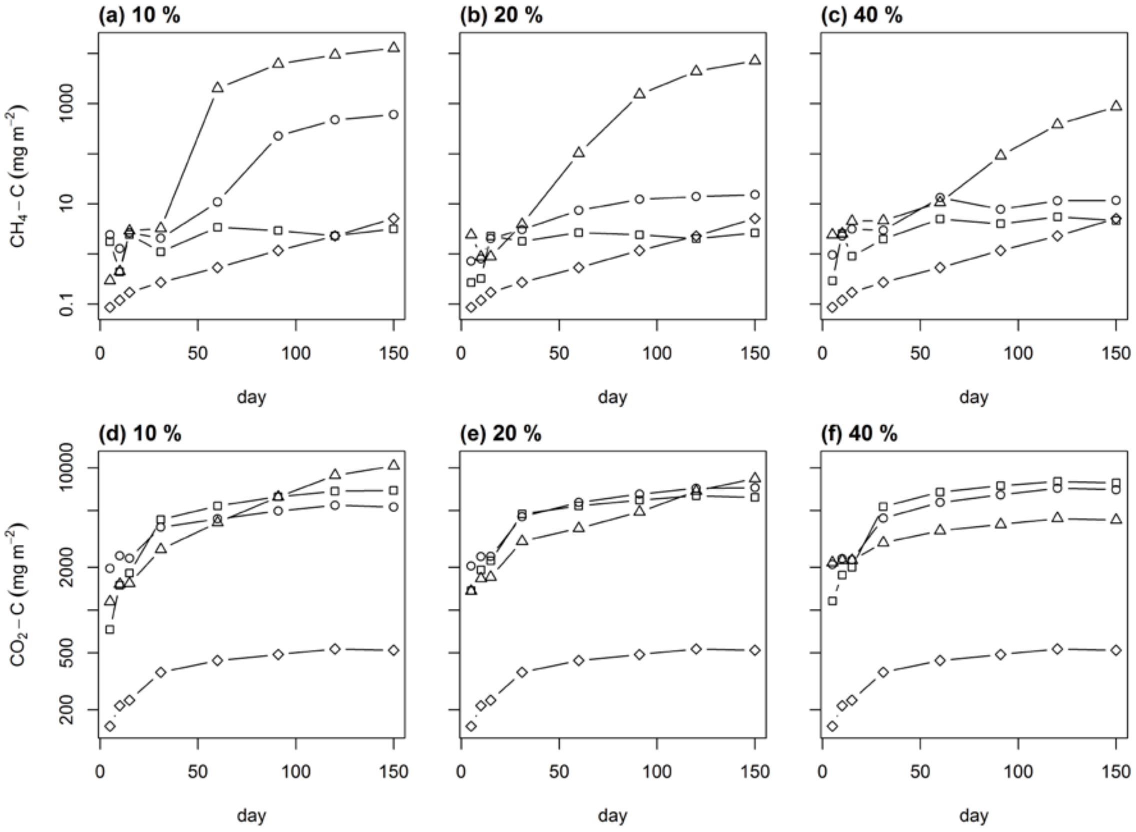

Supplementary Fig. 5: Rates of CH_4 and CO_2 production plateaued after 150 days of incubation. Concentrations of CH_4 (a, b, c) and CO_2 (d, e, f) at 10, 20 and 40% organic matter are shown across measurement days (day 5, 10, 15, 31, 60, 91, 120, 150) as means of the four replicates of non-spiked sediments. The control (CTR) sediments with 0% OM (diamonds; ◊) are plotted in each panel for comparison to TYP (triangles; Δ), CON (circles; ○), and DEC (squares; □) sediments.

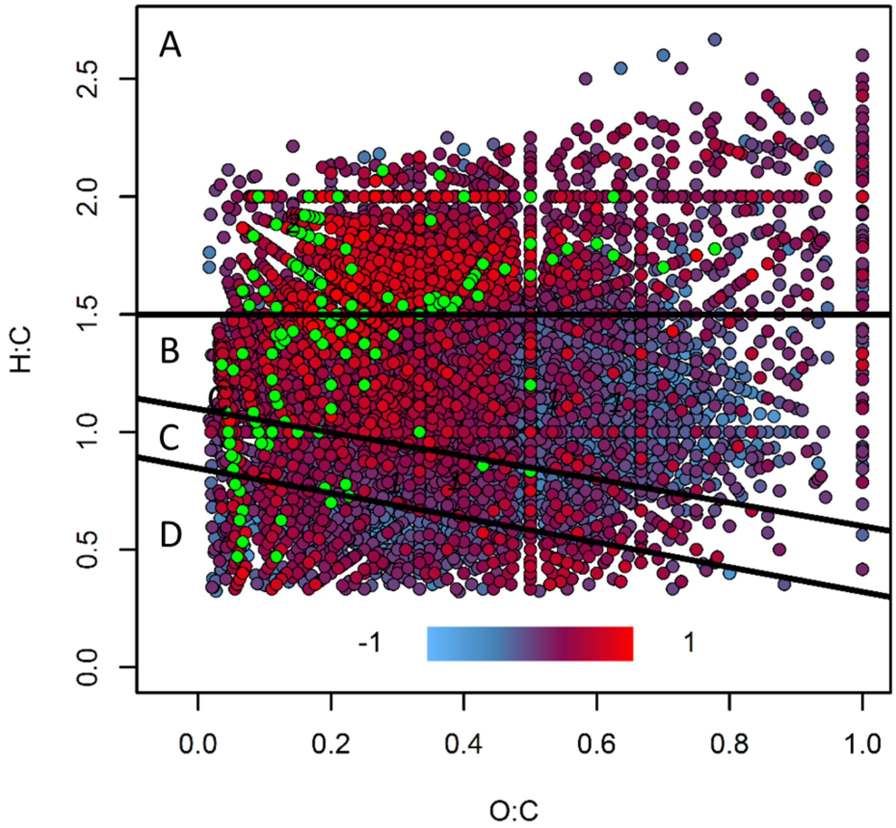

Supplementary Fig. 6: PARAFAC component C5 uniquely correlates with fresher, vascular plant-derived polyphenols, phenolic compounds, and other highly condensed aromatic compounds. Points represent 10,820 individual molecular formulae detected and coloured according to Spearman's rank correlation coefficients between their normalized peak intensity (described in Methods) and proportion of C5 fluorescence across 39 porewater samples. Molecules that are exclusively correlated to C5 and no other fluorescence component with a coefficient ≥ 0.49 are indicated in green. Four molecular compound groups are indicated. The group of molecules in region A comprises saturated and aliphatic compounds (H/C > 1.5). Region B contains highly unsaturated and phenolic compounds (modified aromaticity index AImod < 0.50 and H/C < 1.5). Region C contains polyphenols / aromatic compounds (0.66 ≥ AImod ≥ 0.50). Region D contains polycyclic aromatics (AImod >0.66), including condensed combustion-derived dissolved black carbon. AImod was calculated as in ref. 4 and groupings were determined as in refs 5,6.

References

1. Preston, C. M., Bhatti, J. S. & Norris, C. E. Chemical quality of aboveground litter inputs for jack pine and black spruce stands along the Canadian Boreal Forest Transect Case Study. *Ecoscience* **21,** 202–216 (2014).

2. Bastviken, D., Tranvik, L. J., Downing, J. A., Crill, P. M. & Enrich-Prast, A. Freshwater Methane Emissions Offset the Continental Carbon Sink. *Science,* **331,** 50–50 (2011).

3. Mckenney, D. W., Pedlar, J. H., Lawrence, K., Campbell, K. & Hutchinson, M. F. Beyond traditional hardiness zones: Using climate envelopes to map plant range limits. *Bioscience* **57,** 929–937 (2007).

4. Koch, B. P. & Dittmar, T. Erratum: From mass to structure: An aromaticity index for high-resolution mass data of natural organic matter (Rapid Communications in Mass Spectrometry (2006) 20 (926-932) DOI: 10.1002/rcm.2386). *Rapid Commun. Mass Spectrom.* **30,** 250 (2016).

5. Seidel, M. *et al.* Composition and Transformation of Dissolved Organic Matter in the Baltic Sea. *Front. Earth Sci.* **5,** 1–20 (2017).

6. Kellerman, A. M., Dittmar, T., Kothawala, D. N. & Tranvik, L. J. Chemodiversity of dissolved organic matter in lakes driven by climate and hydrology. *Nat. Commun.* **5,** 3804 (2014).
